# Causal Role of Neural Signals Transmitted From the Frontal Eye Field to the Superior Colliculus in Saccade Generation

**DOI:** 10.3389/fncir.2018.00069

**Published:** 2018-08-28

**Authors:** Masayuki Matsumoto, Ken-ichi Inoue, Masahiko Takada

**Affiliations:** ^1^Division of Biomedical Science, Faculty of Medicine, University of Tsukuba, Tsukuba, Japan; ^2^Graduate School of Comprehensive Human Sciences, University of Tsukuba, Tsukuba, Japan; ^3^Transborder Medical Research Center, University of Tsukuba, Tsukuba, Japan; ^4^Systems Neuroscience Section, Primate Research Institute, Kyoto University, Inuyama, Japan; ^5^Precursory Research for Embryonic Science and Technology (PRESTO), Japan Science and Technology Agency (JST), Kawaguchi, Japan

**Keywords:** frontal eye field, superior colliculus, saccade, optogenetics, macaque monkey

## Abstract

The frontal eye field (FEF) and superior colliculus (SC) are major and well-studied components of the oculomotor system. The FEF sends strong projections to the SC directly, and neurons in these brain regions transmit a variety of signals related to saccadic eye movements. Electrical microstimulation and pharmacological manipulation targeting the FEF or SC affect saccadic eye movements. These data suggest the causal contribution of each region to saccade generation. To understand how the brain generates behavior, however, it is critical not only to identify the structures and functions of individual regions, but also to elucidate how they interact with each other. In this review article, we first survey previous works that aimed at investigating whether and how the FEF and SC interact to regulate saccadic eye movements using electrophysiological and pharmacological techniques. These works have reported what signals FEF neurons transmit to the SC and what roles such signals play in regulating oculomotor behavior. We then highlight a recent attempt of our own that has applied an optogenetic approach to stimulate the neural pathway from the FEF to the SC in nonhuman primates. This study has shown that optogenetic stimulation of the FEF-SC pathway is sufficiently effective not only to modulate SC neuron activity, but also to evoke saccadic eye movements. Although the oculomotor system is a complex neural network composed of numbers of cortical and subcortical regions, the optogenetic approach will provide a powerful strategy for elucidating the role of each neural pathway constituting this network.

## Introduction

The oculomotor system is composed of numbers of cortical and subcortical regions that form a complex neural network. The frontal eye field (FEF) and the superior colliculus (SC) are major components of this system. The roles of the FEF and SC in regulating oculomotor behavior have long been investigated in macaque monkeys in which the oculomotor system is substantially developed. Electrophysiological recording studies have reported that neurons in both the FEF and the SC transmit a variety of signals related to saccadic eye movements, ranging from visual responses evoked by saccadic targets to burst firing before and after the execution of the eye movements (Bizzi, 1968; Schiller and Koerner, 1971; Wurtz and Goldberg, 1971; Mohler et al., 1973; Bruce and Goldberg, 1985). Electrical microstimulation of the FEF or SC elicits saccadic eye movements (Robinson and Fuchs, 1969; Robinson, 1972; Schiller and Stryker, 1972; Bruce et al., 1985), while pharmacological inactivation of either region severely disrupts the eye movements (Hikosaka and Wurtz, 1985; Dias et al., 1995; Sommer and Tehovnik, 1997; Dias and Segraves, 1999). Notably, inactivation of the FEF more severely disrupts saccadic eye movements than that of other cortical oculomotor areas (Sommer and Tehovnik, 1999; Chafee and Goldman-Rakic, 2000). These works suggest the strong causal contribution of the FEF and SC to saccade generation.

Understanding how the brain generates behavior, however, requires more than just analyzing the roles of individual regions. It is critical to determine how particular regions interact with each other and how their interaction contributes to behavior. Since the FEF and SC are mutually connected in a manner that the former projects directly to the latter (Fries, 1984; Komatsu and Suzuki, 1985; Stanton et al., 1988b), while the latter sends signals indirectly to the former via the mediodorsal nucleus of the thalamus (MD; Sommer and Wurtz, 2002, 2004a,b), these regions could interact to regulate saccadic eye movements. Here we first review prior electrophysiological and pharmacological studies that aimed at investigating what signals FEF neurons transmit to the SC and what roles these signals play in regulating oculomotor behavior. Evidence accumulated by these studies indicates that the FEF is largely involved in saccade generation by conveying oculomotor signals to the SC.

The optogenetic methodology that has been used to stimulate signal transmission connecting two given brain regions (Bernstein and Boyden, 2011; Tye and Deisseroth, 2012) allows us to directly address what roles the neural signals transmitted from the FEF to the SC play in regulating saccadic eye movements. Such optogenetic methodology has brought substantial success in modulating behaviors in rodents (Stuber et al., 2011; Tye et al., 2011; Stamatakis and Stuber, 2012; Warden et al., 2012; Ahmari et al., 2013; Miyamoto et al., 2016), and has advanced our understanding of the roles of particular neural circuits in behaviors. After reviewing the electrophysiological and pharmacological studies, we highlight a recent attempt of our own that has applied an optogenetic approach to stimulate the neural pathway from the FEF to the SC in macaque monkeys. This study has shown that optogenetic stimulation of the FEF-SC pathway is sufficiently effective not only to modulate SC neuron activity, but also to evoke saccadic eye movements. The same procedure will be relevant to elucidating other neural network functions as well and will provide significant advances in understanding of brain mechanisms underlying behavioral control in nonhuman primates.

## Roles of the FEF-SC Pathway in Saccade Generation: Electrophysiological and Pharmacological Studies

**Figure 1:**
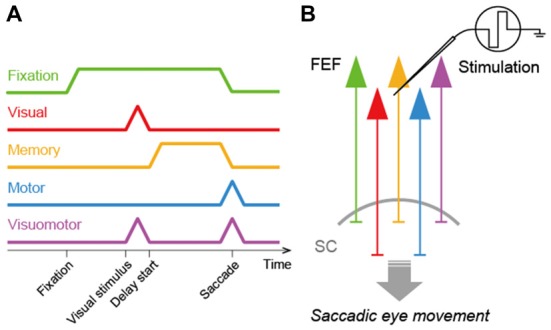
Signal transmission in the frontal eye field-superior colliculus (FEF-SC) pathway. **(A)** Diagram of signals transmitted from the FEF to the SC. The FEF transmits fixation- (green), visual- (red), memory- (orange), motor- (blue) and visuomotor- (purple) related signals to the SC. **(B)** Schema of the FEF-SC pathway. The effect of FEF stimulation on saccadic eye movements is predominantly mediated by this pathway.

In order to consider how the FEF and SC interact to regulate saccadic eye movements, it is crucial to determine what signals FEF neurons transmit to the SC. To address this issue, previous studies identified FEF neurons projecting to the SC and recorded the activity of these neurons in macaque monkeys performing a visually- or memory-guided saccade task (Segraves and Goldberg, 1987; Sommer and Wurtz, 2000, 2001). To identify the SC-projecting FEF neurons, they were antidromically activated by electrical microstimulation of the SC. Sommer and Wurtz (2000) found that the corticotectal neurons transmitted a variety of signals related to saccadic eye movements. These neurons exhibited visual responses to saccadic targets, tonic discharges during the delay period of the memory-guided saccade task, and/or burst firing before and after the execution of the eye movements. In addition, some corticotectal neurons showed discharges during fixation. Thus, the FEF relays visual-, memory-, motor- and even fixation-related signals to the SC (Figure 1). Since all these signals are observed in a broad population of FEF neurons as well, the FEF is likely to provide the SC with non-biased, general signals rather than any specific signals.

In contrast with the above observations, however, Segraves and Goldberg (1987) reported that SC-projecting FEF neurons preferentially transmitted motor-related signals. They found that the corticotectal neurons had little or no response to visual stimuli, but exhibited very strong activity before both visually- and memory-guided saccadic eye movements. Sommer and Wurtz (2000) discussed why these two studies reached the contradictory results. The most critical reason may be related to the topographic distribution of responsive neurons. Although Sommer and Wurtz (2000) found that the corticotectal neurons conveyed a variety of signals, neuronal populations with distinct signals were distributed differently in the FEF. In fact, the neurons with motor-related signals tended to be located in the medial part of the FEF, and tended to project to the caudal level of the SC. If Segraves and Goldberg (1987) focused their recordings on the medial FEF or their stimulations on the caudal SC, this could account for why they observed a larger proportion of motor-related neurons than other neuron types. Consistent with the observations in the study of Sommer and Wurtz (2000); Everling and Munoz (2000) found that the antidromically-identified corticotectal neurons in the FEF transmitted not only motor-, but also visual-related signals during the performance of a pro- or an anti-saccade task.

The FEF sends projections not only to the SC, but also to other oculomotor structures in the brainstem (Stanton et al., 1988b). Therefore, the FEF might contribute to saccade generation via its output pathway to the brainstem that bypasses the SC. To test whether FEF signals transmitted to the SC participate in saccade generation, Hanes and Wurtz (2001) pharmacologically inactivated the SC in macaque monkeys and examined the effect of electrical microstimulation of the FEF on saccadic eye movements in the animals. In their experimental condition, the FEF-SC pathway was disrupted but the FEF-brainstem pathway was kept intact. If the FEF-brainstem pathway is more responsible for saccade generation than the FEF-SC pathway, then the FEF microstimulation is supposed to evoke saccadic eye movements even in SC-inactivated animals. Contrary to this supposition, they found that the SC inactivation eliminated saccadic eye movements evoked by the FEF microstimulation. Notably, this effect was observed when the stimulation site in the FEF and the inactivation site in the SC topographically overlapped. These findings suggest that the FEF-brainstem pathway is not sufficiently operative to generate saccadic eye movements on their own, and that FEF signals transmitted to the SC contribute to saccade generation. It should be noted, however, that bilateral lesions of the SC do not completely disrupt saccade generation. Schiller et al. (1980) reported that monkeys can produce voluntary saccadic eye movements even only 4 days after bilateral SC lesions, whereas paired lesions of the FEF and SC cause drastic deficits in the eye movements. These findings suggest that the FEF may possess functional routes that bypass the SC to generate saccadic eye movements. Such routes might not work in normal animals, but become active in the absence of the SC.

## Roles of The FEF-SC Pathway in Saccade Generation: Optogenetic Approach

As mentioned above, Hanes and Wurtz (2001) showed the causal contribution of the FEF signals transmitted to the SC to saccade generation. However, the FEF not only directly projects to the SC, but also indirectly sends signals to the SC via other brain structures, such as the basal ganglia (for details, see the “Future Directions” section). It remains unclear whether the causal contribution is mediated by the direct or indirect pathway from the FEF to the SC. Optogenetics has provided a powerful tool to address these issues. For instance, neurons in a particular brain region are genetically modified to express channelrhodopsin-2 (ChR2), a blue-light-sensitive cation channel, by injecting a viral vector thereinto. Then, an optical fiber is placed into another region that receives projections from the infected region. Photo-stimulation of the ChR2 expressed on axon terminals induces a synaptic response to evoke signal transmission connecting the two regions. This methodology has brought substantial success in modulating behaviors in rodents (Stuber et al., 2011; Tye et al., 2011; Stamatakis and Stuber, 2012; Warden et al., 2012; Ahmari et al., 2013; Miyamoto et al., 2016), and has advanced our understanding of the roles of particular neural pathways in behaviors. However, the use of this methodology has greatly been restricted to small animals, and its application to primates which have much larger brains than rodents has so far been limited.

**Figure 2:**
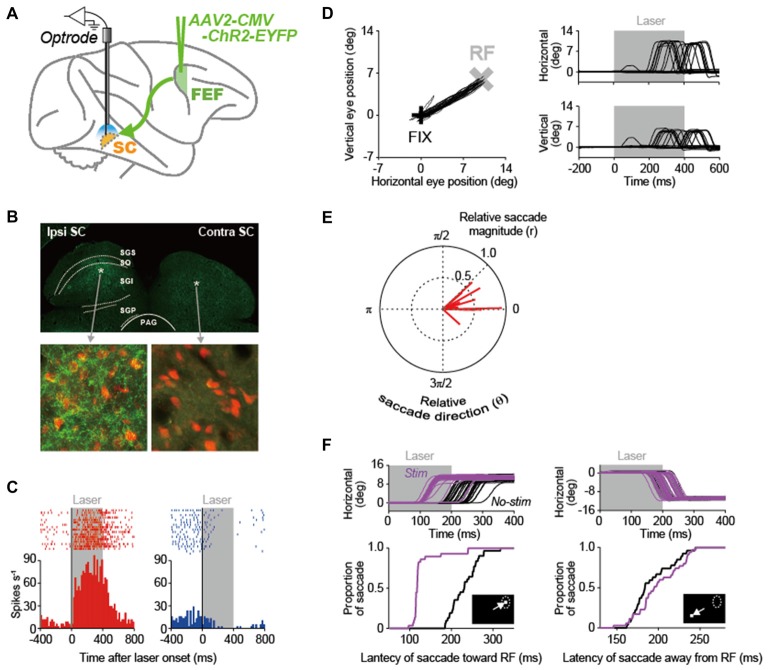
Optogenetic approach to elucidating the role of the FEF-SC pathway in oculomotor behavior. **(A)** Experimental design. In order to deliver the channelrhodopsin-2 (ChR2) gene into FEF neurons, adeno-associated virus type 2 vector (AAV2-CMV-ChR2-EYFP) was injected unilaterally into the FEF. Using optrodes (an optic fiber attached to a recording electrode), 473-nm blue laser light was emitted into the SC and single-unit activity was simultaneously recorded. **(B)** ChR2-EYFP expression in the SC. Top, wide-field immunofluorescent image of a coronal section through the SC. Bottom, immunofluorescence of YFP-expressing axons in the ipsilateral and contralateral SC. NeuN-expressing SC neurons are shown in red. Asterisks in the above panel indicate the locations of the ipsilateral and contralateral images in the SC. PAG, periaqueductal gray; SGI, stratum griseum intermediale; SGP, stratum griseum profundum; SGS, stratum griseum superficiale; SO, stratum opticum. **(C)** Activity of two neuron examples that were excited (left) and inhibited (right) during laser light emission in the ipsilateral SC. Gray areas indicate the period of laser light emission. **(D)** Saccadic eye movements evoked by optical stimulation at a representative site in the ipsilateral SC. Left, trajectories of eye positions. Black and gray crosses indicate the fixation point (FIX) and the center of the response field (RF) at the stimulation site in the SC. The FIX was kept on during laser light emission. Right, horizontal and vertical eye traces. **(E)** Polar plot of the magnitude (r) and direction (θ) of evoked saccades relative to the RF center of the stimulation sites. Red lines indicate the averaged vector of evoked saccades at each stimulation site (*n* = 15). Saccade toward the RF center is denoted with (r, θ) = (1.0, 0). **(F)** Effect of optical stimulation at a representative site in the ipsilateral SC on saccade latency. Top, horizontal eye traces. Bottom, cumulative distribution of the latency of saccades toward the RF (left) and those away from the RF (right). Purple and black curves indicate stimulated and non-stimulated saccades, respectively. Reproduced with permission from Inoue et al. (2015).

In our recent study (Inoue et al., 2015), we made an attempt to apply the optogenetic methodology to the primate oculomotor system that is substantially developed, as compared to the rodent system. Using this methodology, we stimulated the pathway from the FEF to the SC in macaque monkeys and analyzed the causal role of FEF signals transmitted to the SC in saccade generation (Figure 2A). An adeno-associated virus type 2 vector (AAV2-CMV-ChR2-EYFP) was injected unilaterally into the FEF, and, consequently, ChR2-positive axon terminals were observed in the ipsilateral SC, but not in the contralateral SC (Figure 2B). The axon terminals were stimulated by illuminating laser light into the SC. Through the optical stimulation of FEF axon terminals in the ipsilateral SC, many of recorded SC neurons exhibited an excitation that was sustained during laser light emission (left in Figure 2C), while only a few of them displayed an inhibition (right in Figure 2C). Thus, the direct stimulation of FEF signals transmitted to the SC can modulate SC neuron activity.

A pivotal issue of this research is whether the optogenetic methodology could induce behavioral modulations in primates. The optical stimulation of FEF axon terminals often evoked saccadic eye movements toward the response fields (RFs) corresponding to the stimulation sites in the SC (for a representative stimulation site, see Figure 2D). However, as a population, the magnitude of evoked saccadic eye movements was smaller than the eccentricity of the RF center (Figure 2E), suggesting that the intensity of optical stimulation was not enough to evoke full saccades that reach the eccentricity. The stimulation also modulated the latency of saccadic eye movements in a visually-guided saccade task in which the optical stimulation started simultaneously with the onset of a saccadic target that was presented inside or outside the RF. The stimulation decreased the latency of saccades toward the RF (for a representative stimulation site, see left in Figure 2F) and increased it away from the RF (see right in Figure 2F). These data indicate that stimulating the FEF-SC pathway, among the complex oculomotor network, is effective not only to modulate SC neuron activity, but also to initiate saccadic eye movements in macaque monkeys. The same optogenetic approach will be relevant to elucidating the roles of other neural pathways constituting the oculomotor network such as the pathway from the FEF to the lateral intraparietal area (LIP) and that from the LIP to the SC.

## Future Directions

Here, we have introduced the previous studies reporting what signals FEF neurons transmit to the SC and the causal contribution of these signals to saccade generation. However, it is not yet completely clear how the FEF and SC interact to generate saccadic eye movements. For instance, although FEF neurons seem to transmit visual-, memory- and motor-related signals to the SC, it remains to be determined which of these signals are involved in saccade generation. A recent study applied an optogenetic approach to the FEF in macaque monkeys and suppressed the activity of FEF neurons at any given timing in a memory-guided saccade task (Acker et al., 2016). This study found that suppression of FEF neurons during the visual, delay (i.e., memory), or movement periods altered the pattern of saccadic eye movements, suggesting that all of the FEF signals contribute to memory-guided saccades. By applying the circuit-level manipulation to their study, the role of each FEF signal transmitted to the SC in saccade generation will be determined.

The FEF is known to transmit signals related not only to simple saccade generation, but also to several executive functions, such as visual attention (Kodaka et al., 1997; Schall, 2004; Thompson et al., 2005), saccadic response inhibition (Hanes et al., 1998), and working memory (Sommer and Wurtz, 2001; Umeno and Goldberg, 2001). Some of these signals have been shown to be transmitted to the SC (Everling and Munoz, 2000; Sommer and Wurtz, 2001). For instance, Everling and Munoz (2000) identified FEF neurons projecting to the SC using antidromic stimulation in monkeys, and recorded their activity while the monkey was performing a pro- or an anti-saccade task. They found that saccade-related corticotectal neurons were inhibited in the context in which the monkey was required to inhibit a reflexive saccadic eye movement toward a visual stimulus (i.e., anti-saccade task) compared with the context in which the animal was simply required to make the reflexive eye movement (i.e., pro-saccade task). Their findings suggest that the FEF transmits signals involved in the executive control of oculomotor behavior to the SC. It remains to be determined, however, what roles such corticotectal signals play in regulating executive functions. The electrophysiological, pharmacological and optogenetic approaches that we have introduced above will be applicable to address this issue.

Neurons in the FEF exert their effects on the SC not only through the direct projection to the SC, especially to its deeper layers, but also by way of the basal ganglia. The caudate nucleus and the rostral part of the putamen, input stations of the basal ganglia, receive projections from the FEF (Stanton et al., 1988a; Parthasarathy et al., 1992; Neggers et al., 2015) and, in turn, send projections to the substantia nigra pars reticulata (SNr; Parent et al., 1984;François et al., 1987), an output station of the basal ganglia. Then, the SNr sends projections to the deeper layers of the SC (Beckstead et al., 1981; Francois et al., 1984). Thus, the basal ganglia appear to mediate signal transmission from the FEF to the SC. Notably, since the SNr sends GABAergic projections to the SC (Vincent et al., 1978; Di Chiara et al., 1979), the SNr is most likely to exert a strong inhibitory effect on the activity of SC neurons (Hikosaka and Wurtz, 1983). In particular, it has been proposed that the SNr contributes to saccade generation by disinhibiting SC neuron activity (Hikosaka et al., 2000), although the exact role of the FEF-basal ganglia-SC pathway is still unclear. It remains to be determined how the two parallel pathways, i.e., the FEF-SC and FEF-basal ganglia-SC pathways, cooperate in regulating saccadic eye movements.

There are other brain structures than the basal ganglia that connect the FEF and SC. Among them, the cerebellum has attracted attention for its crucial roles in voluntary eye movements (Robinson and Fuchs, 2001). The anatomical organization of cerebro-cerebellar circuits has been established especially by Strick and colleagues (Lynch et al., 1994; Kelly and Strick, 2003; Strick et al., 2009). At least part of the cerebellum projects directly to the SC (May et al., 1990). The cerebellum also communicates with the basal ganglia multisynaptically (Hoshi et al., 2005; Bostan et al., 2010).

We have so far introduced prior attempts that investigated the role of signals transmitted from the FEF to the SC. On the other hand, the SC sends back signals to the FEF via the MD (Sommer and Wurtz, 2002, 2004a,b). It has previously been shown that the SC-MD-FEF pathway transmit signals related to the corollary discharge (or internal copy) of oculomotor command, and that pharmacological inactivation of the MD impairs sequential eye movements consistent with the loss of the corollary discharge (Sommer and Wurtz, 2002, 2004b). Understanding how the entire oculomotor network including the FEF, SC, basal ganglia, cerebellum and MD generates oculomotor behavior is a challenging issue. Optogenetic techniques applicable to primates could have advantages to address this issue.

## Author Contributions

MM conceived of the review topic and all authors wrote the review article.

## Conflict of Interest Statement

The authors declare that the research was conducted in the absence of any commercial or financial relationships that could be construed as a potential conflict of interest.
